# T221. LURASIDONE DISPLAYS ANTIDEPRESSANT AND PRO-COGNITIVE EFFECTS IN THE CHRONIC MILD STRESS MODEL: A ROLE FOR REDOX MECHANISMS AND PARVALBUMIN EXPRESSION

**DOI:** 10.1093/schbul/sby016.497

**Published:** 2018-04-01

**Authors:** Andrea Carlo Rossetti, Maria Serena Paladini, Paola Brivio, Giulia Sbrini, Mariusz Papp, Francesca Calabrese, Raffaella Molteni, Marco Andrea Riva

**Affiliations:** 1 University of Milan; 2 Polish Academy of Sciences, Krakow

## Abstract

**Background:**

Exposure of rodents to chronic stress is able to recapitulate a number of functional alterations that are associated with psychiatric disorders, including anhedonia and cognitive impairment. Stress-based experimental models are also useful to investigate the ability of pharmacological intervention in normalizing such defects as well as the molecular alterations associated with the behavioral phenotype. On these bases, the aim of the present study was to investigate the ability of a chronic treatment with the multi-receptor modulator lurasidone in normalizing behavioral changes produced by chronic mild stress (CMS) in rats. Moreover, we investigated the potential contribution of parvalbumin expression and of redox mechanisms in the alterations brought about by CMS exposure.

**Methods:**

Adult male Wistar rats were exposed to CMS for 2 weeks and sucrose consumption was used to identify rats that were susceptible to the stressful manipulation. Control and CMS-susceptible rats were then randomized to receive chronic vehicle or the multi-receptor modulator lurasidone (3 mg/kg/day) for 5 more weeks, while continuing the stress procedure. Animals were tested for anhedonia, using the sucrose intake test, and for cognitive impairment, using the novel object recognition (NOR) test. Rats were sacrificed at the end of the procedures and the brain regions of interest were dissected and used for the molecular analyses.

**Results:**

Exposure to CMS produced a persistent anhedonic phenotype as well as a significant deficit in the NOR test. Both behavioral abnormalities were normalized by chronic lurasidone treatment. Rats exposed to CMS display a marked and selective reduction in the expression of parvalbumin, which identifies a subpopulation of GABAergic interneurons, in dorsal (but not ventral) hippocampus, an effect that was normalized by chronic lurasidone administration. CMS rats also show a significant up-regulation of (NADPH) oxidase 2 (NOX2), which is critically involved in oxidative stress, as well as a down-regulation of Nrf-2, a master regulator of antioxidant defense. These alterations in dorsal hippocampus were normalized in animals that received chronic lurasidone treatment that was also capable of reducing the levels of Keap-1, an important player that exerts a repressive control over Nrf-2.

**Discussion:**

Our results demonstrate the ability of lurasidone in normalizing anhedonia and cognitive deficits associated with CMS exposure, suggesting its effectiveness on different ‘domains’ (RDoC) that characterize psychiatric disorders. Lurasidone was also able to normalize the effects produced by CMS exposure on parvalbumin expression, possibly through its ability in promoting anti-oxidative mechanisms within the dorsal hippocampus. All in all, these effects may promote resilience toward the alterations produced by adverse environmental conditions, such as stress, which represents a major vulnerability element in the etiology of psychiatric disorders.

